# Association between BDNF and postoperative cognitive dysfunction across surgical stages: a systematic review and meta-analysis

**DOI:** 10.3389/fnagi.2026.1864037

**Published:** 2026-07-23

**Authors:** Danyan Liang, Xiaodong Wang, Yi Qiu

**Affiliations:** 1 School of Public Health, Shanxi Medical University, Taiyuan, Shanxi, China; 2 Inner Mongolia People's Hospital, Hohhot, Inner Mongolia Autonomous Region, China; 3 The Second Affiliated Hospital of Inner Mongolia Medical University, Hohhot, Inner Mongolia Autonomous Region, China

**Keywords:** BDNF, biomarker, intraoperative period, meta-analysis, postoperative cognitive dysfunction

## Abstract

Postoperative cognitive dysfunction (POCD) is a frequent complication after surgery, particularly among older adults, and its underlying mechanisms remain incompletely understood. Brain-derived neurotrophic factor (BDNF) is a key regulator of neuronal survival and synaptic plasticity, and has been implicated in POCD but published findings are inconsistent. We therefore performed a systematic review and meta-analysis to evaluate the association between perioperative BDNF levels and POCD across surgical time points. Following PRISMA guidelines, we searched PubMed, EMBASE, Web of Science, and Scopus were searched for observational studies that reported perioperative BDNF levels in patients with and without POCD. Two reviewers independently extraction data and assessed study quality using the Newcastle-Ottawa Scale. Statistical analyses were conducted in RevMan 5.3, with mean differences (MD) with 95% confidence intervals (CI) were calculated for absolute BDNF levels and perioperative changes (ΔBDNF) at preoperative, intraoperative, and postoperative stages. Six studies including 135 patients with POCD and 247 without POCD met the inclusion criteria. The fixed-effects meta-analysis showed lower intraoperative BDNF levels in the POCD group (MD = −5.62, 95% CI[−8.95, −2.29], *P* = 0.0009; *I*^2^ = 0%). Similarly, intraoperative ΔBDNF was reduced in the POCD group under a fixed-effects meta-model (MD = −9.73, 95% CI[−16.08, −3.38], *P* = 0.003; *I*^2^ = 0%). The subgroup analysis indicated that the type of surgery may contribute to the high heterogeneity observed in 24-h postoperative BDNF and in 24-h postoperative ΔBDNF. These findings suggest that intraoperative BDNF levels and their dynamic changes may serve as preliminary exploratory biomarker for POCD. The intraoperative phase appears to be a critical window for BDNF monitoring, yet prospective cohort studies are urgently needed to externally validate its uesfulness for screening patients at high risk of POCD before clinical implementation. Current evidence remains preliminary, since only six small observational studies were included and substantial statistical heterogeneity was present in multiple postoperative subgroup analyses.

**Systematic review registration:** https://www.crd.york.ac.uk/PROSPERO/view/CRD420251265921.

## 1 Introduction

Postoperative cognitive dysfunction (POCD) manifests as impairments in verbal and visual memory, language processing, visuospatial abilities, attention, and concentration after surgical procedures (Borchers et al., 2021). It is a common postoperative complication, especially in older adults, affecting approximately 25%–40% of patients over 60 years of age and potentially persisting for up to 1 year after surgery (Moller et al., 1998; Monk et al., 2008; Zhao et al., 2024; Rump and Adamzik, 2022; Varpaei et al., 2024), with consequential long-term adverse outcomes in some cases. Multiple factors influence POCD, including educational level, age, type of surgery, anesthetic drugsagents, and nursing interventions (Lam et al., 2026; Sretavan et al., 2026; Melese et al., 2026). The pathophysiology is multifactorial and not fully elucidated. Proposed mechanisms include neuroinflammatory responses, oxidative stress, dysregulated autophagy, neuronal damage, and inadequate neurotrophic support (Bowden et al., 2022; Fu et al., 2024; Liu et al., 2018). Thus, identifying of reliable biomarkers associated with POCD is essential for early diagnosis, prognostic evaluation, as well as for advancing mechanistic understanding.

Multiple circulating biomarkers have been identified to correlate with POCD, including S100β, neuron-specific enolase (NSE), pro-inflammatory cytokines IL-6 and TNF-α. Corresponding meta-analyses have been conducted to synthesize the predictive value of these biomarkers (Peng et al., 2013; Lu et al., 2025; Wang et al., 2023). ADDIN EN.CITE (Peng et al., 2013; Lu et al., 2025; Wang et al., 2023). Brain-derived neurotrophic factor (BDNF) is a neurotrophin that plays a central role in neuronal development, survival, and synaptic plasticity. It regulates neuronal differentiation, growth, and connectivity during development and maintains synaptic function in the adult brain (Kowiański et al., 2018; Wang et al., 2022). Several studies have examined perioperative BDNF expression in patients with POCD across different surgical stages (Miniksar et al., 2021; Knysh et al., 2021; Ruhnau et al., 2023). In addition, there have been some efforts to examine both circulating precursor forms and mature BDNF in relation to postoperative cognitive outcomes (Dong et al., 2025). Despite growing interest, findings regarding the relationship between BDNF levels and POCD remain inconsistent. Some studies have reported a significant negative association between BDNF levels and the occurrence of POCD (Miniksar et al., 2021; Knysh et al., 2021; Miceli et al., 2025; Zhang et al., 2015), whereas others have reported no significant relationship (Ruhnau et al., 2023; Ji et al., 2013). These inconsistencies may arise from varying study design, patient populations, sample sizes, and timing of BDNF measurement. Accordingly, a comprehensive synthesis of the available evidence is warranted.

Here, we performed a systematic review and meta-analysis to clarify the association between perioperative BDNF concentrations and POCD among surgical patients. We aimed to quantitatively compare BDNF levels at predefined preoperative, intraoperative, and postoperative time points, as well as evaluate dynamic perioperative alterations in BDNF. The results are intended to inform clinical practice by identifying potential biomarkers for early detection and risk stratification of POCD.

## 2 Methods and materials

### 2.1 Data sources and search strategy

This review was performed as per the PRISMA guidelines, with the meta-analysis protocol having been registered in PROSPERO (CRD420251265921). The PubMed, EMBASE, Web of Science, and Scopus databases were searched.

The search strategy incorporated combinations of terms related to POCD and BDNF, as follows:

(“Postoperative cognitive Dysfunction” OR “Post-operative Cognitive Impairment” OR “Postoperative Cognitive Disorder” OR “Postoperative Cognitive Complication” OR “Postoperative Cognitive Decline” OR “POCD” OR “Postoperative Neurocognitive Disorder” OR “Delayed Neurocognitive Recovery”) AND (“BDNF” OR “Brain-Derived Neurotrophic Factor” OR “BDNF protein” OR “Bdnf protein”).

### 2.2 Eligibility criteria

Studies meeting the following criteria were included: (1) observational design involving human participants; (2) inclusion of patients diagnosed with POCD; (3) patients undergoing anesthesia; and (4) reporting of BDNF levels before and after surgery. Studies were excluded if they: (1) lacked sufficient or accessible data; (2) were case reports, reviews, commentaries, or letters; (3) involved interventional designs; or (4) did not provide both preoperative and postoperative BDNF measurements.

### 2.3 Study selection and data extraction

Two reviewers independently evaluated titles and abstracts for eligibility. Full-text evaluation was conducted for potentially relevant studies, and disagreements were resolved by discussing with a third reviewer. Data were extracted independently using a standardized data collection form (Table 1). Extracted variables included sample size, study design, type of surgery, anesthetic modality, perioperative BDNF levels, timing of measurements, cognitive assessment tools, and follow-up intervals for postoperative cognitive outcomes. For studies reporting BDNF data as medians with interquartile ranges, values were converted to means and standard deviations using the Statistical Platform for Life Sciences. Changes in BDNF levels (ΔBDNF) during the perioperative period were calculated using preoperative values as the baseline reference.

**Table 1:** Characteristics of studies.

NO.	Author	Country	Year	NOS	Study period	Age	Type of surgery	Type of anesthesia	Total number	Number of POCD (M: F)	Number of nPOCD (M: F)	Test of POCD	Time of POCD	Type of samples	BDNF detection time	AUC
1	Johanna Ruhnau	Germany	2023	7	2018–02/2020-03	≥ 60	Spine Surgery	General anesthesia	44	19 (8:11)	25 (12:12)	Nursing Delirium Screening Scale, Nu-DESC	Three times daily for at least 72 h	Serum	Biosamples were collected at preoperative, perioperative, and two postoperative days.	Not reported
2	Okkes Hakan Miniksar	Turkey	2021	7	2020-01/2020-08	59.4 ± 10.42	CABG	General anesthesia	45	17 (17:0)	28 (23:5)	Montreal Cognitive Assessment (MoCA)	before surgery; 4 days after surgery	Serum	T1: after induction before surgery (baseline), T2: 15 min before aortic cross-clamp removal, T3: 15 min after aortic cross-clamp removal, T4: 4 days after surgery	0.759
3	Vitale Miceli	Italy	2025	8	2019-02/2020-07	61.7 ± 13.29	CABG	General anesthesia	20	9 (0:9)	11 (10:1)	MMSE RBANS	Before surgery;3month after surgery	Plasma	Baseline and 3-month follow-up	Baseline 0.899; 3-month follow-up 0.768
4	Yushan Dong	China	2025	8	2021-10/2022-05	>65	Orthopedic surgery	General anesthesia	86	17 (5:12)	69 (32:37)	MoCA	Before surgery; 7 days after surgery	Serum	BDNF were measured one day before and one day after the surgery, respectively	0.722
5	ZHANG Yuan	China	2015	7	2013-01/2014-01	65–79	Colorectal surgery	General anesthesia	79	19 (13:6)	58 (36:22)	MMSE; visual verbal learning test; digital span test; digital symbol test	1 days before surgery; 7 days after surgery	Serum	24 h before operation and after operation 4 h	Not report
6	Knysh S.V.	Russia	2021	6	Not reported	67–69	CABG	Not reported	110	54 (32:22)	56 (35:21)	MoCA	before surgery; 7 days after surgery	Serum	Before surgery, immediately after surgery, 24 h after surgery and 7 days after surgery	0.974

NOS, Newcastle-Ottawa scale; CABG, coronary bypass surgery; AUC, area under the ROC curve; M, male; F, female.

### 2.4 Quality assessment

Study methodological quality was subject to independent evaluation by two reviewers with the Newcastle-Ottawa Scale (NOS), which evaluates non-randomized studies based on selection, comparability, and outcome/exposure. Scores ranged from 0 to 9, with scores greater than 7 being indicative of high methodological quality. Any discrepancies were resolved via consensus.

### 2.5 Statistical analysis

Meta-analyses were performed using RevMan 5.3. Effect sizes were expressed as mean difference (MD) and 95% confidence intervals (CIs). Funnel plots were constructed to visually evaluate publication bias.Statistical heterogeneity was assessed using the Chi-square test and the *I*^2^ statistic. A fixed-effect model was applied when heterogeneity was low (*I*^2^ < 50%), whereas a random-effects model was used when heterogeneity was substantial (*I*^2^ ≥ 50%).

### 2.6 Sensitivity and subgroup assessment

To explore the factors that might contribute to the expected variability, we conducted subgroup analyses and sensitivity analyses based on the following study characteristics: type of surgery, type of assessment scales and type of sample. All of them are recognized as potential effect modifiers across included studies.

## 3 Results

### 3.1 Literature search and study characteristics

**Figure 1:**
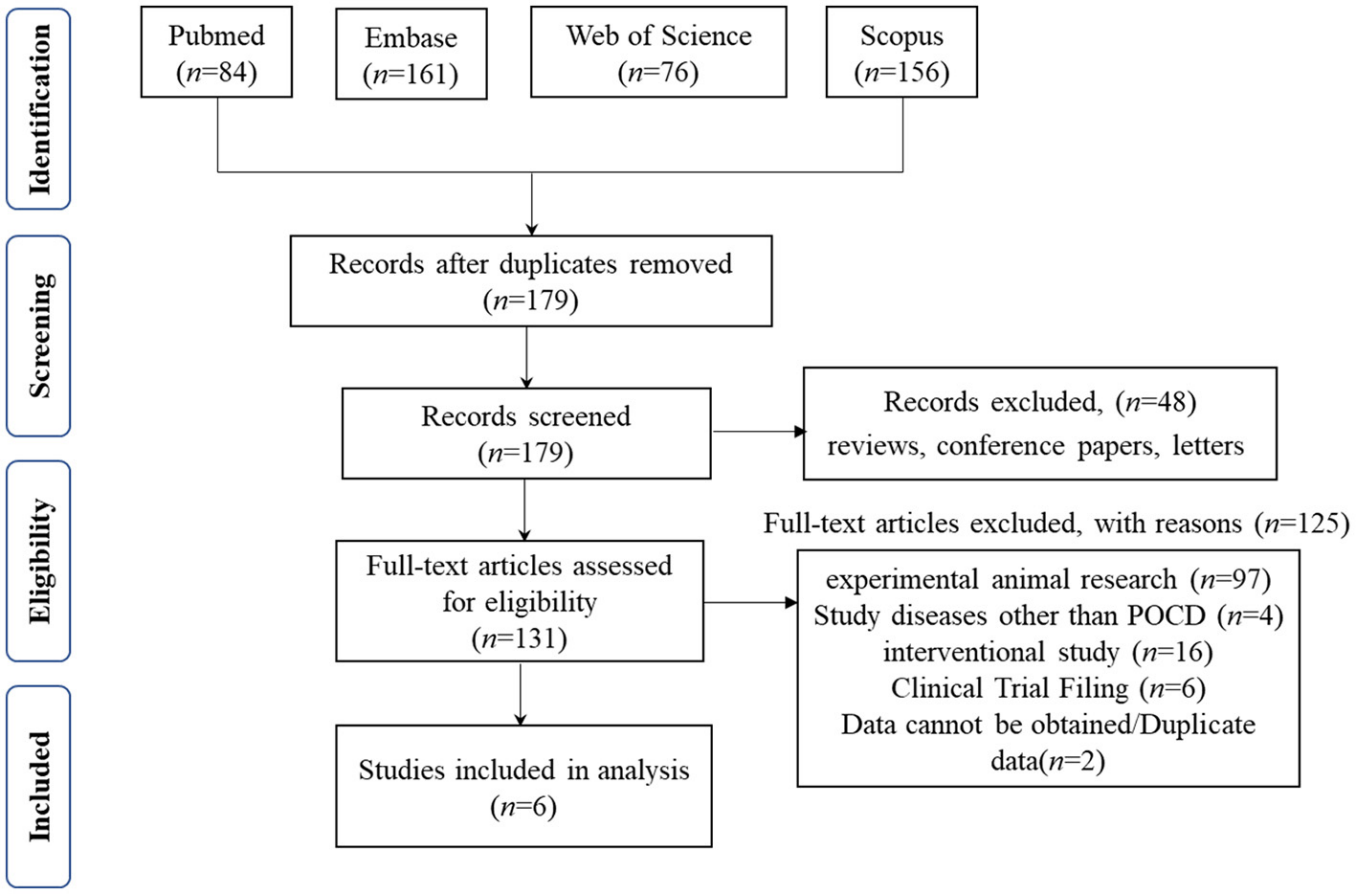
PRISMA flow diagram.

A total of 477 potentially relevant records were initially retrieved from multiple databases using predefined keywords and MeSH terms. After removal of duplicates and preliminary screening of titles and abstracts, 179 articles remained for further evaluation. Of these, 131 full-text articles were evaluated for eligibility, and 48 were excluded for not meeting the inclusion criteria. Ultimately, 6 studies were included in the final meta-analysis. Study selection is detailed in Figure 1.

The 6 included studies (Miniksar et al., 2021; Knysh et al., 2021; Ruhnau et al., 2023; Dong et al., 2025; Miceli et al., 2025; Zhang et al., 2015) included 135 patients diagnosed with POCD and 247 individuals without POCD. Geographically, two studies were conducted in China, while one study each originated from Germany, Turkey, Italy, and Russia. All enrolled participants were older than 60 years. Regarding surgical types, three studies (60.0%) involved patients undergoing coronary artery bypass grafting (CABG), whereas the remaining studies included spinal, orthopedic, and colorectal procedures. In terms of cognitive assessment, half of the studies (3/6, 50.0%) utilized a single evaluation tool, while the other half employed multiple assessment scales. All studies conducted POCD evaluations at least twice across different postoperative time points. For BDNF measurement, three studies (50.0%) collected samples during both preoperative and intraoperative phases, and three studies (50.0%) assessed BDNF at more than three perioperative time points. Additionally, four studies (66.7%) reported receiver operating characteristic (ROC) curve analyses. Detailed characteristics of the included studies are presented in Table 1.

### 3.2 Quality assessment

Study quality was evaluated using NOS scores, with a maximum possible score of 9. Overall, the included studies demonstrated acceptable methodological quality. Five studies achieved scores from 7 to 8, indicating high quality, while one study received a score of 6 (Table 1).

### 3.3 Association between BDNF levels and POCD

**Figure 2:**
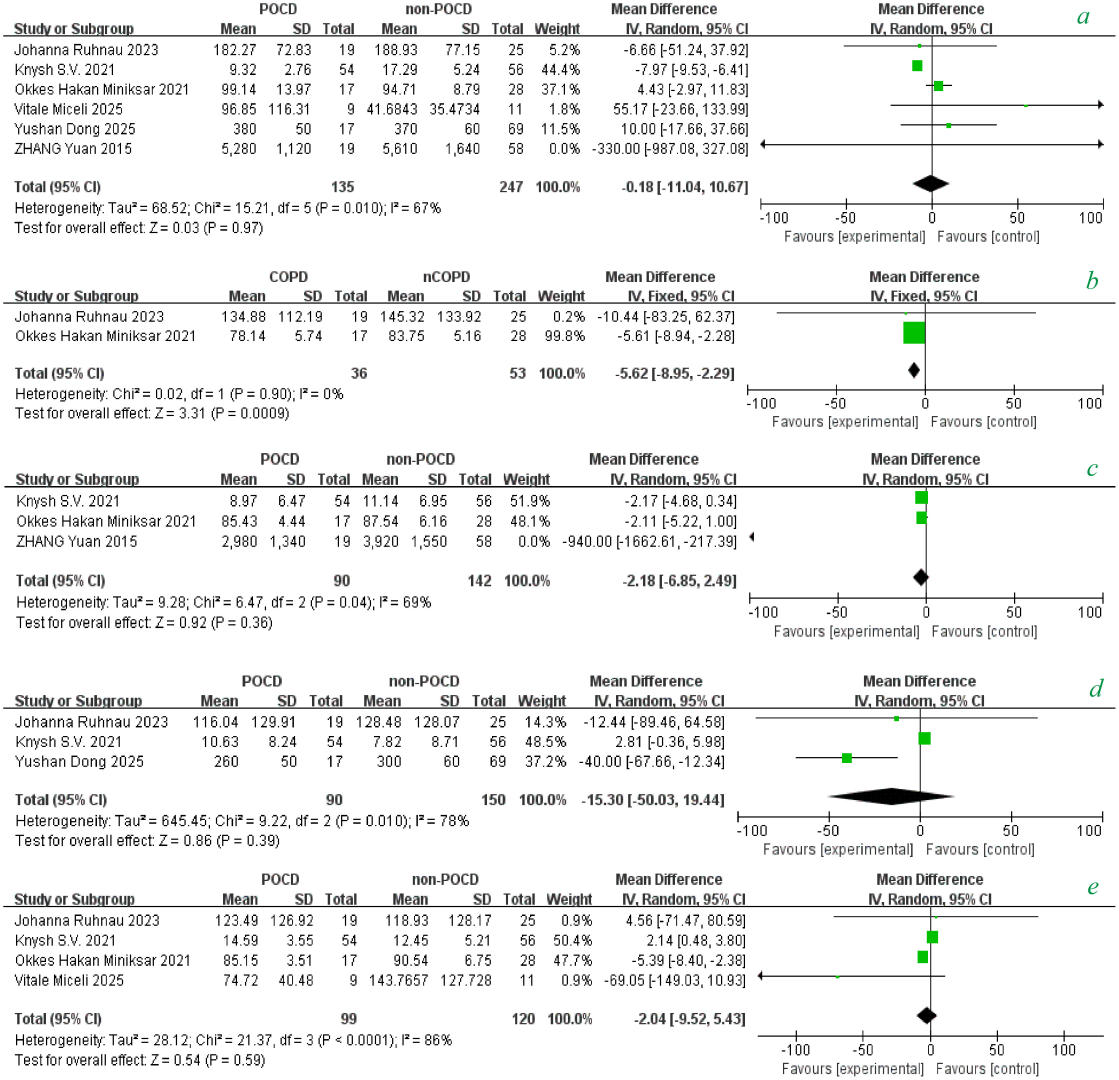
Meta-analysis of the association between BDNF levels and POCD across surgical stages. **(a)** preoperative BDNF; **(b)** intraoperative BDNF; **(c)** immediate postoperative BDNF; **(d)** 24 h postoperative BDNF; **(e)** 7-day postoperative BDNF.

Meta-analysis revealed no significant difference in preoperative BDNF levels when comparing patients who developed POCD and those who did not (MD = −0.18; 95% CI[−11.04, 10.67]; *P* = 0.97; *I*^2^ = 61%; Figure 2a). In contrast, intraoperative BDNF levels were significantly lower in the POCD group compared with the non-POCD group using a fixed-effects model. (MD = −5.62; 95% CI[−8.95, −2.29]; P = 0.0009; *I*^2^ = 0%; Figure 2b). No significant differences were observed between the two groups at postoperative time points, including immediately after surgery (MD = −2.18; 95% CI[−6.85, 2.49]; *P* = 0.36; *I*^2^ = 69%), 24 h postoperatively (MD = −15.30; 95% CI[−50.03, 19.44]; *P* = 0.39; *I*^2^ = 78%), and 7 days postoperatively (MD = −2.04; 95% CI[−9.52, 5.43]; *P* = 0.59; *I*^2^ = 86%; Figures 2c–e).

We assessed result robustness using a leave-one-out sensitivity analysis. Excluding the study by Knysh et al. (2021). reduced *I*^2^ for preoperative BDNF to 0%, while the overall conclusion remained unchanged (Supplementary Table 1). For 24 h postoperative BDNF, removal of the study by Zhang et al. (2015). also lowered *I*^2^ to 0% and yielded a pooled estimate showing decreased BDNF in the POCD group vs. the non-POCD group. Subgroup analyses mainly showed lower BDNF in POCD than in non-POCD among patients undergoing non-cardiac surgery (Supplementary Table 2). Likewise, surgical method appeared to be a major source of heterogeneity for the 24 h postoperative ΔBDNF (Supplementary Table 2). In the 7-day postoperative BDNF analysis, excluding the studies by Knysh et al. (2021) and by Miniksar et al. (2021) reduced heterogeneity.

### 3.4 Association between changes in BDNF and POCD

**Figure 3:**
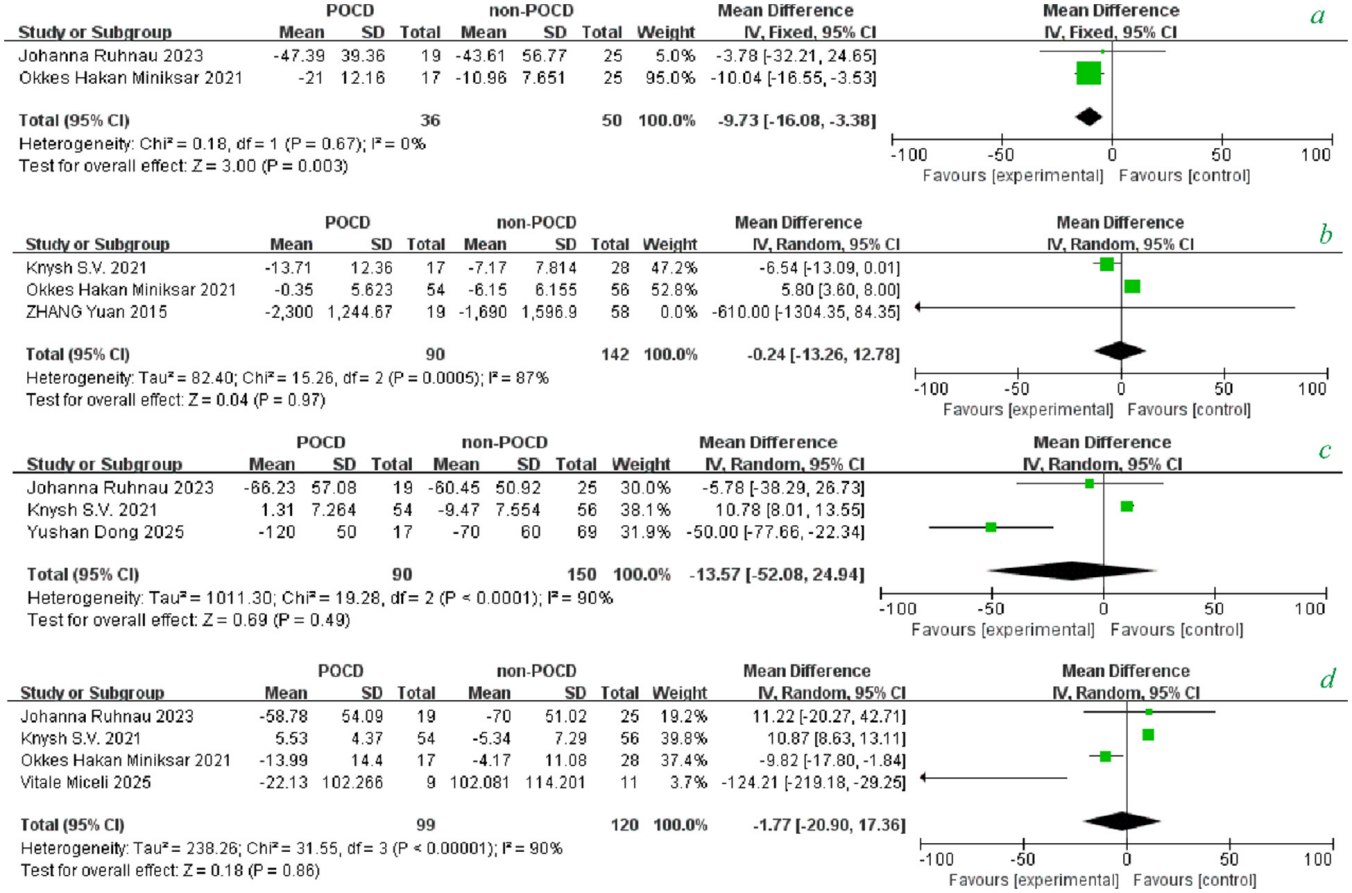
Meta-analysis of the association between perioperative changes in BDNF and POCD. **(a)** Intraoperative ΔBDNF; **(b)** Immediate postoperative ΔBDNF; **(b)** 24 h postoperative ΔBDNF; **(d)** 7-day postoperative ΔBDNF.

Using preoperative BDNF levels as the baseline, perioperative changes in BDNF (ΔBDNF) were calculated for each time point. Intraoperatively, ΔBDNF was lower in the POCD groupthan non-POCD group (MD = −9.73; 95% CI[−16.08, −3.38]; *P* = 0.003; *I*^2^ = 0%; Figure 3a). However, no significant differences in ΔBDNF were identified at other time points, including immediately after surgery (MD = −0.24; 95% CI[−13.26, 12.78]; *P* = 0.97; *I*^2^ = 87%), 24 h postoperatively (MD = −13.57; 95% CI[−52.08, 24.94]; *P* = 0.49; *I*^2^ = 90%), and 7 days postoperatively (MD = −1.77; 95% CI[−20.90, 17.36]; P = 0.86; *I*^2^ = 90%; Figures 3b–d).

### 3.5 Publication bias and selective reporting

**Figure 4:**
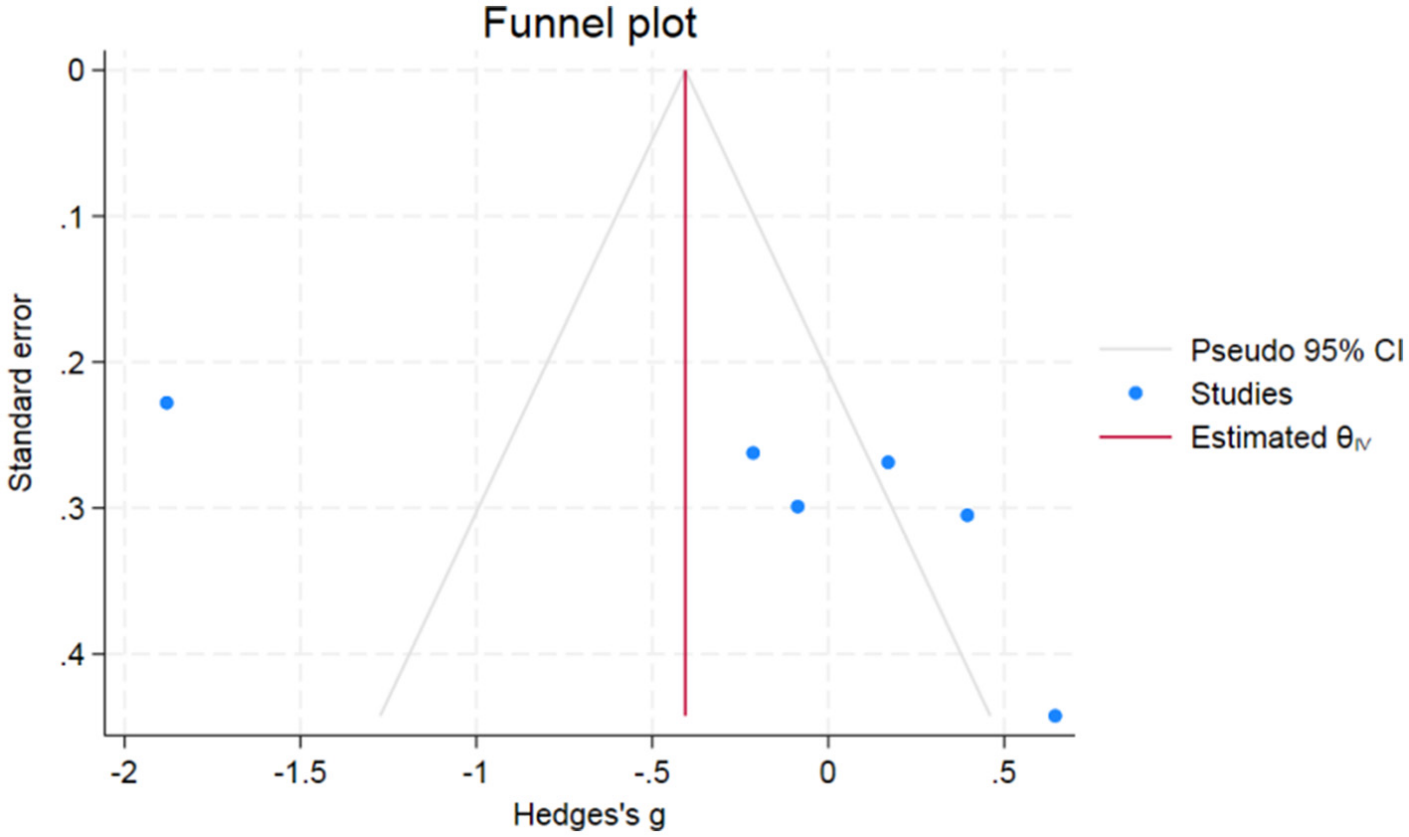
Funnel plot analysis.

We assessed publication bias using funnel plot analysis and observed no marked asymmetry (Figure 4). The Egger test corroborated this finding (*Z* = 1.90; *P* = 0.0579). Nevertheless, determining the presence of subtle publication bias and selecting an appropriate method to address it remain challenging.

## 4 Discussion

POCD is characterized by subtle impairments in memory and broader cognitive domains following anesthesia and surgery, affecting patients of all ages but occurring more often in older adults (Suraarunsumrit et al., 2024; Anand et al., 2024). The studies included in this meta-analysis mainly enrolled individuals aged ≥60 years, suggesting that the sample reflects the demographic at highest risk. Despite extensive research, the pathophysiology of POCD, as well as standardized nomenclature and diagnostic criteria, remain incompletely defined (Parker et al., 2022; Alam et al., 2018). Accordingly, identifying reliable biomarkers with diagnostic or prognostic value and potential therapeutic relevance remains a priority in perioperative medicine.

In recent years, neuroinflammation has emerged as a central mechanism in POCD (Chen et al., 2020). Surgical stress and anesthesia provoke heightened inflammatory responses and reducneurotrophic support, both of which contribute to cognitive decline. Increasing evidence indicates that epigenetic alterations, including DNA methylation of inflammatory mediators (e.g., TNF-α, IL-6, IL1β) and neuronal genes such as BDNF, as well as histone modifications (e.g., H3K9 and H4K12 acetylation), may serve as candidate biomarkers for POCD (Rump and Adamzik, 2022). BDNF, a key neurotrophin family member, regulates neuronal survival, differentiation, synaptic plasticity, and memory formation by activating its high-affinity receptor TrkB (Lin et al., 2021). It is crucial for long-term potentiation, dendritic branching, and synaptic spine density, particularly within the hippocampus (Wang et al., 2022). The relationship between BDNF and POCD is multifaceted and involves several signaling pathways. Previous studies have reported that BDNF demonstrates diagnostic potential for POCD, with AUC values from 0.722 to 0.974, indicating a reasonable capacity to discriminate between POCD and non-POCD cases. Disruption of BDNF-TrkB signaling has been implicated in cognitive dysfunction in disorders such as Alzheimer's disease and chronic pain (Zhang et al., 2025; Shademan et al., 2025). Mechanistically, reduced BDNF expression in hippocampal CA1 glutamatergic neurons impairs synaptic plasticity and neuronal excitability, thereby contributing to POCD (Wu et al., 2024). Conversely, BDNF overexpression preserves synaptic integrity, enhances neuronal activity, and attenuates surgery-related cognitive deficits.

Another critical issue is the timing of biomarker assessment. Current evidence suggests that systemic inflammatory and neuronal responses are rapidly initiated after surgical insult and may approach peak levels within 6–12 h following major procedures (Peng et al., 2013; Ruhnau et al., 2023; Lin et al., 2014; Johnsson et al., 2000; Sentürk et al., 2025). Although the temporal dynamics vary among biomarkers, this early window is considered particularly informative. In the present analysis, we compared BDNF levels between POCD and non-POCD groups across multiple perioperative stages. The results demonstrated a reduction in intraoperative BDNF levels in patients who developed POCD (MD = −5.62; 95% CI:[−8.95; −2.29]; *P* < 0.05). In contrast, measurements taken before the operation, immediately after the operation, 24 h postoperatively, and 7 days postoperatively exhibited instability. Subgroup analysis identified the type of surgery as the primary source of heterogeneity. A similar pattern was observed for ΔBDNF. Specifically, intraoperative ΔBDNF was significantly lower in the POCD group (MD = −9.73; 95% CI:[−16.08; −3.38]; *P* < 0.05), whereas no meaningful differences were detected at other time points. These findings consistently indicate that the intraoperative period represents the phase during which divergence in BDNF dynamics between groups is most pronounced.

The perioperative interval, especially the early postoperative phase, constitutes a critical window for biomarker evaluation and therapeutic intervention. Wang et al. (2021) reported that perioperative sleep disturbances influence POCD via BDNF-related mechanisms. Xue et al. (2022) identified the postoperative BDNF/proBDNF ratio as a key determinant of cognitive outcomes, and a potential therapeutic target. Given BDNF's central role in neural function, interventions that enhance BDNF signaling may help prevent or mitigate POCD (Palasz et al., 2020; Allen et al., 2013). Han et al. (2023). demonstrated that remote ischemic preconditioning (RIPC) raises perioperative BDNF levels and improves cognition. Wu et al. (2024) emphasized the early postoperative period as an optimal window for targeting the SIRT1/BDNF signaling axis. Fineberg et al. (2025) showed that delayed fluoxetine administration can extend the therapeutic window for BDNF modulation beyond this immediatephase. Similarly, Wang et al. (2024) found that early postoperative regulation of microRNA (miR-206-3p) alters BDNF expression and supports cognitive recovery. Beyond pharmacological strategies, non-pharmacological interventions, such as cognitive training and physical exercise, have improved cognitive performance, potentially by upregulating BDNF (Li et al., 2025). Efforts to target neuroinflammation and BDNF-related pathways are also under active investigation as therapies for POCD (Pan et al., 2025; Ruhnau et al., 2025). Overall, although the perioperative period is clearly critical, inconsistencies remain regarding the optimal timing for biomarker monitoring and intervention. Future studies should refine these temporal windows to enhance early detection and therapeutic efficacy in POCD management.

## 5 Limitations

This study has several limitations. First, the number of eligible studies was relatively small, and in some analyses only a limited number of datasets could be pooled, potentially affecting the reliability of the formal assessment of publication bias. Second, several included studies had modest sample sizes and employed heterogeneous methods for outcome assessment, thus, larger cohorts studies are needed to address population-specific confounding. Third, substantial heterogeneity between studies was observed, although a random-effects model was applied to account for this variability, residual heterogeneity may still influence the robustness of the findings. Further, most included studies were observational in design. Given that this study aimed to examine associations between BDNF and POCD, observational designs provide an appropriate foundation for hypothesis generation.

Key directions for future research include standardizing the timing of BDNF sampling, harmonizing diagnostic criteria for POCD establishing a larger multicenter prospective cohort, and validating the clinically relevant threshold of BDNF across different surgical types.

## 6 Conclusions

In conclusion, intraoperative BDNF concentrations declined in the POCD group, indicating BDNF's potential as a biomarker for early detection of POCD. The intraoperative period therefore offers a critical window for monitoring dynamic BDNF changes. Correlations between BDNF and POCD across other surgical phases, however, remain inconsistent. Prospective cohort studies are urgently needed to validate BDNF's effectiveness for preoperative screening of patients at high risk for POCD before clinical implementation.

## Data Availability

The raw data supporting the conclusions of this article will be made available by the authors, without undue reservation.
