# Supplementary material for: Association between BDNF and postoperative cognitive dysfunction across surgical stages: a systematic review and meta-analysis
Source: Front Aging Neurosci. 2026 Jul 23;18:1864037. doi: 10.3389/fnagi.2026.1864037 (PMC13443294; doi:10.3389/fnagi.2026.1864037)
Supplement: Supplementary file 1 [file Table_1.docx]

Leave-one-out meta-analysis

| Study | *n* | | MD(95% CI ) | | *P* | I^2^ |
| --- | --- | --- | --- | --- | --- | --- |
|  | POCD | non-POCD |  |  |  |  |
| Preoperative BDNF | | | | | | |
| Omitting Johanna Ruhnau 2023 | 116 | 222 | 0.42 | (-11.29, 12.13) | 0.94 | 74% |
| Omitting Knysh S.V. 2021 | 81 | 191 | 4.88 | (-2.15, 11.91) | 0.17 | 0% |
| Omitting Okkes Hakan Miniksar 2021 | 118 | 219 | -3 | (-16.51, 10.52) | 0.66 | 20% |
| Omitting Vitale Miceli 2025 | 126 | 236 | -1.35 | (-11.71, 9.01) | 0.80 | 69% |
| Omitting Yushan Dong 2025 | 118 | 178 | -1.48 | (-13.09, 10.12) | 0.80 | 71% |
| Omitting ZHANG Yuan 2015 | 116 | 189 | -0.08 | (-10.97, 10.81) | 0.99 | 72% |
| 24 h postoperative BDNF | | | | | | |
| Omitting Knysh S.V. 2021 | 36 | 86 | -398.59 | (-1306.67, 509.48) | 0.39 | 85% |
| Omitting Okkes Hakan Miniksar 2021 | 73 | 114 | -398.62 | (-1306.63, 509.40) | 0.39 | 85% |
| Omitting ZHANG Yuan 2015 | 71 | 84 | -2.15 | (-4.10, -0.19) | 0.03 | 0% |
| 7-day postoperative BDNF | | | | | | |
| Omitting Johanna Ruhnau 2023 | 80 | 95 | -2.14 | (-9.85, 5.56) | 0.59 | 91% |
| Omitting Knysh S.V. 2021 | 45 | 64 | -5.46 | (-8.47, -2.46) | 0.0004 | 20% |
| Omitting Okkes Hakan Miniksar 2021 | 82 | 92 | 2.11 | (0.45, 3.77) | 0.01 | 34% |
| Omitting Vitale Miceli 2025 | 90 | 109 | -1.46 | (-8.60, 5.68) | 0.69 | 89% |
| 24 h postoperative △BDNF | | | | | | |
| Omitting Johanna Ruhnau 2023 | 71 | 125 | -17.99 | (-77.47, 41.49) | 0.55 | 95% |
| Omitting Knysh S.V. 2021 | 36 | 94 | -28.75 | (-72.05, 14.55) | 0.19 | 76% |
| Omitting Yushan Dong 2025 | 73 | 81 | 10.66 | (7.90, 13.42) | 0.32 | 0% |
| 7-day postoperative △BDNF | | | | | | |
| Omitting Johanna Ruhnau 2023 | 80 | 95 | -5.35 | (-27.60, 16.89) | 0.64 | 94% |
| Omitting Knysh S.V. 2021 | 45 | 64 | -14.95 | (-51.19, 21.28) | 0.42 | 72% |
| Omitting Okkes Hakan Miniksar 2021 | 82 | 92 | -4.5 | (-41.00, 32.00) | 0.81 | 74% |
| Omitting Vitale Miceli 2025 | 90 | 109 | 2.78 | (-14.67, 20.22) | 0.76 | 92% |
