# Supplementary material for: Association between BDNF and postoperative cognitive dysfunction across surgical stages: a systematic review and meta-analysis
Source: Front Aging Neurosci. 2026 Jul 23;18:1864037. doi: 10.3389/fnagi.2026.1864037 (PMC13443294; doi:10.3389/fnagi.2026.1864037)
Supplement: Supplementary file 2 [file Table_2.docx]

Subgroup analyses

|  | Group | *n* | | MD(95% CI ) | | *P* | *I*^2^ |
| --- | --- | --- | --- | --- | --- | --- | --- |
|  |  | POCD | non-POCD |  |  |  |  |
| Preoperative BDNF | | | | | | | |
| Surgery type | cardiac surgery | 80 | 95 | -0.83 | (-13.54, 11.88) | 0.90 | 84% |
|  | non-cardiac surgery | 55 | 152 | 4.94 | (-18.55, 28.43) | 0.68 | 0% |
| Type of assessment scales | Non-MoCA | 47 | 94 | 7.14 | (-31.59, 45.88) | 0.72 | 29% |
|  | MoCA | 88 | 153 | -0.81 | (-12.06, 10.45) | 0.89 | 83% |
| Type of sample | serum | 126 | 236 | -1.35 | (-11.71, 9.01) | 0.80 | 69% |
| 24 h postoperative BDNF | | | | | | | |
| Surgery type | cardiac surgery | 54 | 56 | 2.81 | (-0.36, 5.98) | 0.08 | - |
|  | non-cardiac surgery | 36 | 94 | -36.85 | (-62.89, -10.81) | 0.006 | 0% |
| Type of assessment scales | Non-MoCA | 19 | 25 | -12.44 | (-89.46, 64.58) | 0.75 | - |
|  | MoCA | 71 | 125 | -16.3 | (-58.01, 25.41) | 0.44 | 89% |
| Type of sample | serum | 90 | 150 | 2.23 | (-0.91, 5.38) | 0.16 | 78% |
| 7-day postoperative BDNF | | | | | | | |
| Surgery type | cardiac surgery | 80 | 95 | -2.14 | (-9.85, 5.56) | 0.59 | 91% |
|  | non-cardiac surgery | 19 | 25 | 4.56 | (-71.47, 80.59) | 0.91 | - |
| Type of assessment scales | Non-MoCA | 28 | 36 | -30.38 | (-85.49, 24.72) | 0.28 | 41% |
|  | MoCA | 71 | 84 | -1.52 | (-8.89, 5.86) | 0.69 | 95% |
| Type of sample | serum | 90 | 109 | -1.46 | (-8.60, 5.68) | 0.69 | 89% |
| 24 h postoperative △BDNF | | | | | | | |
| Surgery type | cardiac surgery | 54 | 56 | 10.78 | (8.01, 13.55) | <0.001 | - |
|  | non-cardiac surgery | 36 | 94 | -28.75 | (-72.05, 14.55) | 0.19 | 76% |
| Type of assessment scales | MoCA | 71 | 125 | -17.99 | (-77.47, 41.49) | 0.55 | 95% |
| Type of sample | serum | 90 | 150 | -13.57 | (-52.08, 24.94) | 0.49 | 90% |
| 7-day postoperative △BDNF | | | | | | | |
| Surgery type | cardiac surgery | 80 | 95 | -5.35 | (-27.60, 16.89) | 0.64 | 94% |
|  | non-cardiac surgery | 19 | 25 | 11.22 | (-20.27, 42.71) | 0.48 | - |
| Type of assessment scales | Non-MoCA | 28 | 36 | -48.78 | (-180.64,83.07) | 0.47 | 86% |
|  | MoCA | 71 | 84 | 0.89 | (-19.37, 21.16) | 0.93 | 96% |
| Type of sample | serum | 90 | 109 | 2.78 | (-14.67, 20.22) | 0.76 | 92% |
